# Physicochemical Characteristics, Antioxidant Capacity, and Antimicrobial Activity of Stingless Bee Honey from Malaysia: *Heterotrigona itama*, *Lophotrigona canifrons*, and *Tetrigona binghami*

**DOI:** 10.3390/foods14060995

**Published:** 2025-03-14

**Authors:** En Ruth Tiang, Lingyun Han, Fuliang Hu

**Affiliations:** College of Animal Sciences, Zhejiang University, Hangzhou 310058, China; 22117129@zju.edu.cn (E.R.T.); 22117054@zju.edu.cn (L.H.)

**Keywords:** stingless bee honey, physicochemical characteristics, antioxidant, antimicrobial

## Abstract

The composition and bioactivity of honey are influenced by its botanical, geographical, and entomological origins. This study investigates the physicochemical characteristics, antioxidant capacity, and antimicrobial activity of stingless bee honey (SBH) produced by three Malaysian stingless bee species: *Heterotrigona itama*, *Lophotrigona canifrons*, and *Tetrigona binghami*. The moisture content ranges from 25.44% to 40.36%, while the honey color varies from light amber to dark amber. The fructose, glucose, and sucrose contents range from 5.45 to 16.91 g/100 g, 3.85 to 19.64 g/100 g, and undetectable to 2.47 g/100 g, respectively. Trehalulose is present at a level of 15.42 to 43.75 g/100 g, with *L. canifrons* honey exhibiting the highest trehalulose concentration. All samples show low 5-HMF content and no detectable diastase activity. *T. binghami* honey has the lowest pH, highest electricity conductivity and acidity, and exhibits the strongest antimicrobial activity against *Staphylococcus aureus* and *Escherichia coli*. *H. itama* honey exhibits the highest antioxidant potential based on ABTS, FRAP, and DPPH assays. Among the three species, *L. canifrons* honey contains the highest total phenolic content. These findings provide valuable insights into the unique properties of SBH, supporting further research, quality assessment, and the development of international standards.

## 1. Introduction

Stingless bees (Insecta: Hymenoptera: Apidae: Meliponini), named for their lack of a functional sting, are social insects inhabiting tropical and subtropical regions. Compared to honeybees, over 600 species of stingless bees have been recorded across more than 60 named genera, demonstrating remarkable diversity [1]. Despite their small size, stingless bees play an essential role in pollination, contributing to ecosystem stability and plant diversity [2]. Their honey, pollen, and propolis have been reported to possess medicinal properties, including antioxidant and antimicrobial activities [3].

Unlike honeybees, stingless bees store nectar and pollen in cerumen pots instead of wax combs [4]. Their honey undergoes a unique fermentation process, in which bacteria and yeast consume sugars and convert them into alcohol and acetic acid. This process contributes to the sweet-sour taste of stingless bee honey and may also enrich it with biological compounds [5,6]. Due to its distinct flavor and high nutritional value, stingless bee honey is often sold at a higher price, leading to adulteration cases [7,8].

In recent years, numerous studies have examined the antioxidant and antimicrobial properties of stingless bee honey, commonly using indicators such as total phenolic content (TPC), DPPH radical scavenging activity, FRAP, and antimicrobial assays against pathogens like *Escherichia coli* and *Staphylococcus aureus* [9,10,11,12]. Some studies have even shown that stingless bee honey exhibits superior antioxidant and antimicrobial activities compared to *Apis mellifera* honey [13,14,15].

Beyond these properties, stingless bee honey has demonstrated various biological activities. *Melipona subnitida* honey, produced from *Mimosa quadrivalvis*, has been found to be rich in polyphenolic compounds, providing neuroprotective, hepatoprotective, and anti-lipid peroxidation effects in obese rats [16]. Additionally, stingless bee honey exhibits anti-inflammatory effects by reducing the levels of nitrogen oxides (NOx), tumor necrosis factor-α (TNF- α), interleukin-6 (IL-6), monocyte chemoattractant protein-1 (MCP-1), active interleukin-12 (IL-12p70), and interferon- γ (IFN- γ) [17]. Furthermore, its anti-cancer activity potential has been demonstrated through the inhibition of cell proliferation and induction of early apoptosis in malignant glioma cell lines [18]. Other reported biological activities include anti-aging activity [19], antidiabetic effects [20], promoting wound healing [21], and antiproliferative effects [22]. Given these promising bioactivities, stingless bee honey has garnered increasing scientific interest.

Malaysia is rich in stingless bee resources, with 38 identified species. However, only *Geniotrigona thoracica*, *Heterotrigona itama*, *Lepidotrigona terminata*, and *Tetragonula leviceps* have been commercialized [23]. Stingless bee honey in Malaysia, commonly known as “kelulut honey”, is valued for its complex composition, with water and sugars being its primary components [24]. Despite its unique flavor and the high diversity of stingless bee species in Malaysia, consumer awareness of this honey remains limited, possibly due to its lower production volume and market exposure. On average, Malaysian stingless bee colonies produce approximately 4 kg of honey per year, significantly lower than the yield of honey bees [23].

Stingless bee honey exhibits distinct physicochemical properties such as high moisture content, electrical conductivity, acidity, and low pH [25]. As a result, it often fails to meet the honey standards set by the International Honey Commission (IHC) [26]. Furthermore, trehalulose has been identified as a major component in stingless bee honey, distinguishing it from honeybee honey [27]. These factors have led to the ongoing development of national standards for stingless bee honey in countries including Malaysia [28], Australia and New Zealand [29], and China [30].

In this study, honeys from *Heterotrigona itama*, *Lophotrigona canifrons*, and *Tetrigona binghami* were selected for analysis. *Heterotrigona itama* honey is the most commercially available and widely distributed in Malaysia. *Lophotrigona canifrons*, despite being confined to specific regions and exhibiting more aggressive behavior [31,32], produces a notably high volume of honey with a unique aroma. In contrast, *Tetrigona binghami* honey stands out due to its higher moisture and mineral content among stingless bee honeys [33,34].

Given these backgrounds and characteristics, this study aims to characterize the physicochemical properties, antioxidant capacity, and antimicrobial properties of these stingless bee honeys. Our research seeks to provide a scientific basis for defining appropriate quality parameters tailored to stingless bee honey. By evaluating its physicochemical and bioactive properties, this study will contribute to establishing standardized quality benchmarks essential for its commercial production and regulatory frameworks.

## 2. Materials and Methods

### 2.1. Stingless Bee Honey (SBH) Samples

A total of 30 SBH (Table 1) produced by *Heterotrigona itama*, *Lophotrigona canifrons*, and *Tetrigona binghami* were harvested from different regions in Malaysia. All raw honey samples were freshly collected from stingless bee honey pots using a small pump, and then were filtered and stored at approximately −20 °C until analysis. The honeys had a multifloral origin, with the plant sources listed below.

### 2.2. Chemical Reagents

Fructose (≥99%), glucose (≥99.8%), and sucrose (≥99%) were obtained from Beijing Solepol Technology Co., Ltd. (Beijing, China); trehalulose (≥90%) was obtained from UK Biosynth Carbosynth (Compton, Berkshire, UK); sodium hydroxide, iodine, potassium iodide, sodium acetate, glacial acetic acid, sodium chloride, and soluble starch were obtained from Nanjing Aijer Chromatography Technology Co., Ltd. (Nanjing, China); Folin–Ciocalteu phenol was obtained from Shanghai Yuanye Bio-Technology Co., Ltd. (Shanghai, China); 1,1-diphenyl-2-picrylhydrazyl radical (DPPH) and water-soluble vitamin E (Trolox) were obtained from Merck Life Science Technology Co., Ltd. (Nantong, China); the Total Antioxidant Capacity Assay Kit (FRAP method) and the Total Antioxidant Capacity Assay Kit (ABTS method) were obtained from Beyotime Biotechnology Co., Ltd. (Shanghai, China); gallic acid and catalase were obtained from Shanghai Aladdin Biochemical Technology Co., Ltd. (Shanghai, China); nutrient agar was obtained from Beijing Solarbio Science & Technology Co., Ltd. (Beijing, China); 7.5% sodium chloride broth was obtained from Guangdong Huankai Microbial Sci. & Tech. Co., Ltd. (Shanghai, China); LB broth was obtained from Hangzhou Microbial Reagent Co., Ltd. (Hangzhou, China); *Staphylococcus aureus* (ATCC 29213) and *Escherichia coli* (ATCC 25922) were obtained from Shanghai Beinuo Biotechnology Co., Ltd. (Shanghai, China); and sodium carbonate, ethanol, phenol, and other chemical reagents were purchased from China National Pharmaceutical Group Chemical Reagent Co., Ltd. (Shanghai, China). All other reagents used in the experiments were of analytical grade.

### 2.3. Analytical Methods to Determine Physicochemical Characteristics of SBH

#### 2.3.1. Sugar Content

A 5% (*w*/*v*) solution of SBH was prepared in distilled water and filtered through a 0.22 μm nylon membrane into a 1 mL injection vial. Fructose, glucose, and sucrose contents were determined according to the standard methods of the International Honey Commission [35]. A high-performance liquid chromatography-refractive index detector (HPLC-RID, Shimadzu, Kyoto, Japan) was used for analysis, with a ZORBAX NH_2_ column (250 × 4.6 mm, 5 μm, Aligent, Palo Alto, CA, USA). The temperature of both the column and the detector was set to 40 °C, with a mobile phase consisting of acetonitrile/water (70:30 *v*/*v*) at a flow rate of 1 mL/min and an injection volume of 20 μL. Trehalulose was determined using the method described by Zheng et al. [36]. Briefly, HPLC-RID and a Hi-Plex column (300 mm × 7.7 mm, 8 μm, Agilent, Palo Alto, CA, USA) were used. The column and detector temperatures were set to 50 °C, with water as the mobile phase at a flow rate of 0.5 mL/min and an injection volume of 10 μL.

#### 2.3.2. Moisture Content, Color Analysis, and Acidity

The moisture content, color, and acidity of SBH were analyzed using the Rules for the Inspection of Honey for Import and Export of China Standards (SN/T 0852-2012) [37]. Moisture content was determined using a NAR-2T Abbe Refractometer (ATAGO Scientific Instruments Co., Ltd., Guangzhou, China) by measuring the refraction index at 40 °C. Honey color was assessed using a HI96785 Honey Colorimeter (Hanna Instruments, Hangzhou, China), ensuring that all samples were homogeneous and free of air bubbles. Acidity was determined by dissolving 10 g of SBH in 75 mL of boiled and cooled water. Subsequently, 2 to 3 drops of phenolphthalein indicator were added. The solution was titrated using a Titrette (Brand, Germany) with a standardized sodium hydroxide solution until a persistent pink color remained for at least 10 s.

#### 2.3.3. Electrical Conductivity (EC)

The electrical conductivity of SBH was determined following the China National Standards (GB/T 18932.15-2003) [38]. A 20 g sample of SBH was dissolved in water and diluted to a final volume of 100 mL. Measurements were performed using a FE38 Conductivity Meter (Mettler Toledo Technology Co., Ltd., Hangzhou, China), and the results were expressed in μs/cm.

#### 2.3.4. pH

The pH of SBH was measured according to the China National Standards (GB 5009.237-2016) [39]. An appropriate amount of SBH was diluted with water, and the pH was measured using a calibrated FE28 pH Meter (Mettler Toledo Technology Co., Ltd., Hangzhou, China).

#### 2.3.5. Diastase Activity

The diastase activity of SBH was determined using the spectrophotometric method according to the China National Standards (GB/T 18932.16-2003) [40]. Briefly, 5 g of SBH was mixed with 15 mL of distilled water and 2.5 mL of acetate buffer solution. The mixture was then diluted to a final volume of 25 mL, with the addition of 1.5 mL of sodium chloride. Next, 5 mL of the starch solution, 10 mL of the sample solution, and 10 mL of the iodine solution were placed in a water bath at 40 °C for 15 min. Every 5 min, 1 mL of the sample mixture was added to 10 mL of the iodine solution. The absorbance was measured at 660 nm using a UV2700i UV–Vis Spectrophotometer (Shimadzu Corporation, Kyoto, Japan). The result was measured until the absorbance dropped below 0.235.

#### 2.3.6. Content of 5-Hydroxymethylfurfural (5-HMF)

The 5-HMF content of SBH was measured according to the China National Standards (GB/T 18932.18-2003) [41]. Briefly, 10 g of SBH was thoroughly mixed with 10 mL of methanol and then diluted with distilled water to a final volume of 1000 mL. The sample was filtered through a 0.45 μm filter membrane before analysis. Detection and quantification were determined using a Liquid Chromatograph 20ADXR (Shimadzu Corporation) equipped with a UV detector and a Diamonsil-C18 column (250 mm × 4.6 mm, 5 μm). The column temperature was maintained at 30 °C, and the injection volume was 10 μL. The mobile phase consisted of methanol and water (10:90) at a flow rate of 1 mL/min, and the detection wavelength was 285 nm.

### 2.4. Analytical Methods to Determine Antioxidant Activity of Stingless Bee Honey

#### 2.4.1. Determination of Total Phenolic Content (TPC)

The total phenolic content of the stingless bee honey samples was determined using the Folin–Ciocalteu method as described by Wilczyńska [42] with some modifications. Briefly, 10 µL of a 0.2 g/mL honey solution was thoroughly mixed with 50 µL of 0.2 mol/L Folin–Ciocalteu reagent and allowed to stand for 5 min. Subsequently, 40 µL of a 0.075 g/mL Na_2_CO_3_ solution and 100 µL of distilled water were added. After incubation in the dark for 2 h, the absorbance was measured at 760 nm using a Multiskan Sky full-wavelength microplate reader (Thermo Fisher Scientific Co., Ltd., Vantaa, Finland). A standard curve was generated using gallic acid at concentrations ranging from 0.04 to 0.28 mg/mL. The results were expressed as gallic acid equivalents per gram of honey, with the unit µg GAE/g.

#### 2.4.2. 2,2-Diphenyl-1-Picrylhydrazyl (DPPH) Assay

The determination of DPPH radical-scavenging activity was conducted according to the method of Bueno-Costa et al. [43] with slight modifications. In this process, 100 µL of a 0.2 g/mL honey solution was mixed with 100 µL of a 0.08 mg/mL DPPH ethanol solution and incubated in the dark at room temperature for 30 min. The absorbance was measured at 517 nm using a Multiskan Sky full-wavelength microplate reader (Thermo Fisher Scientific Co., Ltd.). A standard curve was constructed using Trolox at concentrations ranging from 0.01 to 0.07 mmol/L. The results were expressed as Trolox equivalent per 100 g of honey, with the unit mg TE/100 g.

#### 2.4.3. 2,2′-Azino-Bis (3-Ethylbenzothiazoline-6-Sulfonic Acid) (ABTS) Assay

The antioxidant capacity of honey samples was determined using the ABTS method with a Total Antioxidant Capacity Assay Kit (Beyotime Biotechnology Co., Ltd., Shanghai, China). The ABTS working solution was prepared by mixing equal volumes of ABTS solution and oxidizing reagent solution, followed by incubation in the dark for 12–16 h and dilution with PBS before use. A 10 mM Trolox standard solution was diluted to concentrations from 0.1 to 1.2 mM to construct a standard curve. Then, 200 µL of ABTS working solution was mixed separately with 10 µL of honey solution and 10 µL of Trolox standard solution in a 96-well plate, with a blank control included. The mixture was incubated at room temperature for 6 min, and absorbance was measured at 734 nm using a Multiskan Sky full-wavelength microplate reader (Thermo Fisher Scientific Co., Ltd.). The results were expressed as Trolox equivalents (TE) per kilogram of honey, in mmol TE/kg.

#### 2.4.4. Ferric Reducing Antioxidant Power (FRAP) Assay

The antioxidant capacity of honey samples was determined using the FRAP method with a Total Antioxidant Capacity Assay Kit. A 100 mM FeSO_4_ solution was diluted to concentrations from 0.15 to 1.5 mM to construct a standard curve. A 5 µL aliquot of a 0.2 g/mL honey solution was mixed with 180 µL of FRAP working solution and incubated in the dark at 37 °C for 5 min. The absorbance was measured at 593 nm using a Multiskan Sky full-wavelength microplate reader (Thermo Fisher Scientific Co., Ltd.). The results were expressed as FeSO_4_ equivalents per kilogram of honey, in mmol FeSO_4_/kg.

### 2.5. Analytical Methods to Determine Antibacterial Activity of Stingless Bee Honey

#### 2.5.1. Agar Diffusion Assay

A 7.5% (*w*/*v*) sodium chloride broth and nutrient agar were prepared and sterilized at 120 °C for 20 min. The sodium chloride broth was used to activate the bacterial strains, which were incubated at 37 °C with shaking at 200 rpm overnight. The optical density (OD) of the bacterial culture was measured at 600 nm and the suspension was diluted to 1 × 10^7^ CFU/mL for use.

The nutrient agar was poured into Petri dishes and allowed to solidify. A 100 µL aliquot of the bacterial suspension was then spread onto the agar surface. Wells with an 8 mm diameter were created using a sterile puncher and the agar plugs were removed. The bottom of each well was sealed before adding test solutions. A 50% (*w*/*v*) honey solution, a 50% (*w*/*v*) honey solution with hydrogen peroxide solution, a 10% phenol solution (as a positive control), and sterile water (as negative control) were prepared. Then, 100 µL of each solution was added to the wells, and the plates were incubated at 37 °C for 18 h. Each experiment was performed in triplicate. The diameter of the inhibition zone was measured using a vernier caliper (mm).

#### 2.5.2. Broth Microdilution Assay

This experiment was adapted from the method of Deng et al. [44] with modifications. The bacterial activation procedure was the same as described above. LB broth was prepared, and the honey samples were diluted to concentrations of 50%, 25%, 20%, 12.5%, 10%, 6.25%, 5%, 3.125%, 2.5%, and 1.25% (*w*/*v*). Each diluted honey sample was mixed with the bacterial suspension at a ratio of 9:1. Positive controls consisted of bacterial suspensions without honey, while negative controls contained sterile honey solution. The mixtures were incubated at 37 °C for 18 h, and absorbance at 600 nm was measured using a microplate reader.

The minimum inhibitory concentration (MIC) assay was initially conducted to evaluate antibacterial activity. However, due to the natural cloudiness of the honey, reliable MIC results could not be obtained, consistent with a previous report by Majkut et al. [45]. Instead, the minimum bactericidal concentration (MBC) was determined. After incubation, 10 µL of each honey-bacterial solution that showed no visible microbial growth was dropped onto nutrient agar plates, which were then incubated at 37 °C for 24 h. The lowest concentration at which no bacterial colonies were observed was recorded as the MBC.

### 2.6. Statistical Analysis

Data were analyzed using Microsoft Excel and Statistical Package for Social Science (SPSS). Results are expressed as the mean ± standard deviation. Analysis of variance (ANOVA) was performed to assess differences among the three types of stingless bee honey. Post hoc comparisons among honey samples were analyzed using Tukey’s test at a significance level of *p* < 0.05. Pearson’s correlation analysis was conducted to assess relationships among different parameters, with all statistical tests performed as two-tailed analyses at a significance level of *p* < 0.05.

## 3. Results and Discussion

### 3.1. Physicochemical Characteristics of SBH

The physicochemical characteristics of Malaysian SBH are summarized in Table 2. The moisture content of SBH ranged from 25.44% to 40.36%, which is relatively high compared to honeybee honey. This increased moisture content is likely influenced by Malaysia’s tropical rainforest climate, characterized by high humidity and substantial rainfall [46]. Among the studied samples, *T. binghami* honey had a significantly higher moisture content than the other SBHs. This aligns with the results of Wong et al. [34] who also reported higher moisture content of *T. binghami* honey compared to *H. itama* honey. However, our results contrast with those of Melia et al. [47], who reported a considerably lower moisture content (23.86%) in *T. binghami* honey from Indonesia.

The color of Malaysian SBH ranged from 31 to 150 mm, corresponding to light amber to dark amber, with *H. itama* honey being the darkest. Pita-Calvo and Vázquez [48] indicated that darker honey usually displayed stronger antioxidant activity.

Sugar analysis revealed that trehalulose was the predominant sugar across all 30 SBH samples, with concentrations ranging from 15.42 to 43.74 g/100 g. *L. canifrons* honey exhibits the highest trehalulose content (38.13 ± 7.38 g/100 g), followed by *H. itama* honey (33.59 ± 7.12 g/100 g) and *T. binghami* honey (20.81 ± 3.52 g/100 g). Notably, this study is the first to report the physicochemical parameters of *L. canifrons* honey.

Previous studies have shown that trehalulose content in SBH varies widely (6.97 to 49.08 g/100 g), with *Geniotrigona thoracica* honey containing the highest levels [7,36,49]. Trehalulose was first identified by Fletcher et al. [27] as a major component of stingless bee honey. This disaccharide is considered a potential sucrose alternative due to its low sweetness (60% of sucrose) [50] and low glycemic index (GI) value [51]. Zhang et al. [52] suggested that the trehalulose formation may be related to the enzymatic activity in the hypopharyngeal glands of the stingless bee.

Among the studied samples, *H. itama* honey exhibited the highest fructose content (11.28 ± 3.59 g/100 g). Zawawi et al. [7] reported a higher mean fructose content for *H. itama* honey (16.23 ± 6.12 g/100 g) and a lower content for *G. thoracica* honey (4.11 ± 2.63 g/100 g). In contrast, *T. binghami* honey exhibits the highest glucose concentration (17.09 ± 3.54 g/100 g), significantly higher than that in *H. itama* honey (11.50 ± 5.00 g/100 g) and *L. canifrons* honey (5.49 ± 1.40 g/100 g). All SBH samples contained low sucrose levels (ND-2.47 g/100 g), with no significant differences among groups. Similarly, Chinese SBH also contained low sucrose levels (ND-0.43 g/100 g) [30], while studies by Zawawi et al. [7] reported the absence of sucrose in Malaysian and Australian SBH.

5-Hydroxymethylfurfural (5-HMF), an indicator for evaluating honey freshness, is affected by temperature and storage duration [53]. In our study, no significant differences in 5-HMF content were observed among the three Malaysian SBH groups. *L. canifrons* honey exhibited the widest 5-HMF range (ND-5.40 mg/kg). These findings align with those of Zheng et al. [30], who demonstrated that the 5-HMF content in Chinese SBH ranges from ND to 9.64 mg/kg. However, Mwangi et al. [54] reported a significantly higher 5-HMF content (22.77 mg/kg) in Kenya SBH. Additionally, geographical origin has been identified as a key factor influencing 5-HMF content, emphasizing that multiple interacting factors contribute to its variation [55].

Diastase activity is commonly used to determine the freshness of honey [56]. In this study, diastase activity was not detected in any of the SBH samples. This finding is consistent with previous studies such as that of Zheng et al. [30], who reported that none of the 89 Chinese SBH samples exhibited detectable diastase activity. Similarly, Chuttong et al. [57] observed diastase activity levels ranging from ND to 4.9 °Gothe in SBH while Biluca et al. [58] found that only 6 of 33 SBH samples exhibited detectable diastase activity.

It is important to note that diastase activity in SBH varies among different stingless bee species. For instance, Cardona et al. [59] reported that *Tetragonisca angustula* honey exhibited the highest diastase activity (10–15 DN), whereas honey from other stingless bee species showed significantly lower levels, ranging from 1.5 to 6.0 DN. According to Bogdanov et al. [60], the low diastase activity in SBH may be attributed to botanical sources and the developmental characteristics of the bee’s gland. Similarly, Pasias et al. [61] reported that honeydew honey exhibits lower diastase activity compared to blossom honey. This finding is relevant to our study, as *Acacia mangium*, a potential nectar source, is classified as honeydew honey [62]. Additionally, environmental factors such as the high temperature and humidity typical of rainforest climates may influence diastase activity in SBH, as high temperatures are known to decrease diastase activity in honey [63]. Further investigation is required to better understand the factors influencing diastase activity levels in SBH.

SBH is generally characterized by lower pH, and higher acidity, and EC depends on the species. In this study, *T. binghami* honey exhibited the lowest pH (2.83), the highest acidity (515.72 meq/kg), and the highest EC (1005.28 μs/cm), which was supported by Sharin et al. [33] and Wu et al. [64]. Conversely, the pH values of Malaysian SBH in this study were lower than those reported for SBH from Mexico and Guatemala [65], while EC values remained within the same range.

Since the current standard for SBH drafted in Malaysia is based on dehydrated stingless bee honey [28], it may not be suitable for raw SBH. Although Malaysia proposed an SBH standard in 2017 [66], the accumulation of recent experimental data underscores the need for its revision to better reflect the latest findings and market demands.

#### Correlation Coefficients Between Physicochemical Parameters

The correlation coefficients between physicochemical parameters are as shown below (Table 3). Notably, the moisture content of SBH showed a significant positive correlation with EC and acidity. The fermentation of stingless bee honey, which produces ethanol and carbon dioxide, likely explains the relationship between acidity and moisture [67]. Trehalulose content strongly correlates with pH but shows negative correlations with moisture content, EC, and acidity. Additionally, pH displayed significant negative correlations with moisture, EC, and acidity. In our study, the strongest correlation was observed between EC and acidity. This aligns with the findings of Avila et al. [25], who reported a close association between these two parameters in honey. Meanwhile, 5-HMF showed no significant correlation with other physicochemical parameters, and honey color correlated only with EC, aligning with the findings of de Sousa et al. [68].

### 3.2. Antioxidant Activity

The antioxidant activity of SBH was evaluated using four methods, and the results are presented in Table 4. Among the samples, *L. canifrons* honey exhibited the highest total phenolic content (TPC), ranging from 531.89 to 730.97 µg GAE/g. While phenolic compounds are known contributors to honey’s antioxidant properties [43], TPC alone may not fully explain the overall antioxidant activity. Notably, *H. itama* honey displayed the strongest antioxidant activity based on the DPPH, ABTS, and FRAP assays, suggesting that other bioactive compounds such as carotenoids, vitamin E, trehalulose, and amino acids also contribute to its antioxidant potential [48,50,54].

Compared to previous studies on *H. itama* honey, our study revealed significantly higher TPC than those reported by Ranneh et al. [69] and Zawawi et al. [7]. According to Shamsudin [70], the botanic origins of honey strongly influence its TPC, which can range from 610.47 to 1140.492 µg GAE/g, explaining the differences observed among studies.

Honeydew honey, produced by bees from the excretions of plant-sucking insects or the secretions of plant tissue [62], generally exhibits stronger antioxidant activity compared to nectar honey due to its higher content of bioactive compounds [71,72,73]. In our study, while all samples were categorized as multifloral, *A. mangium* was identified as one of the potential botanical origins. This fast-growing and widely cultivated tree species in Southeast Asia is an extrafloral nectary plant, with nectaries located on the adaxial side of the basal part of the leafstalk or phyllode [74,75]. The secretion of these nectaries is influenced by the surrounding humidity [74].

Previous research has shown that *A. mangium* honey is rich in phenolic compounds, including naringenin, catechin, kaempferol, benzoic acid, and trans-cinnamic acid, which contribute to its beneficial effects [76]. Among them, naringenin exerts protective effects against metabolic diseases through its antioxidant activity and modulation of key signaling pathways (PI3K/Akt/Nrf2, NF-kB, and MAPK) [77], while catechin plays an important role in its protective effects against cardiovascular diseases, cancer, and other degenerative conditions [78]. These findings highlight the crucial role of phenolic compounds in determining honey’s antioxidant potential.

However, not all SBHs exhibit the same level of antioxidant activity. In our study, *T. binghami* honey exhibited significantly lower antioxidant activity compared to *H. itama* honey, likely due to the reduced presence of antioxidant compounds. Our findings align with those of Melia et al. [47], who reported lower a TPC in *T. binghami* honey than in *H. itama* honey. However, this contrasts with the findings of Wong et al. [34] and Wu et al. [64], suggesting that variations in antioxidant activity may arise from differences in honey composition. Indeed, honey samples from different stingless bee species have exhibited diverse antioxidant profiles. For instance, honey from *Meliponula (Axestotrigona) ferruginea* and *Meliponula (Axestotrigona) togoensis* exhibited a higher TPC (819.2–875.6 µg GAE/g) than those observed in our studies but demonstrated lower FRAP values (1.10–1.27 mmol FeSO_4_/kg) [14]. These variations may be attributed to differences in bee species, geographical location, experimental methodologies, and botanical origins.

#### Correlation Coefficients Between Antioxidant Assays

A significant positive correlation was observed among all antioxidant assays. Among them, DPPH and FRAP exhibited the strongest correlation, with a coefficient of 0.849 (*p* < 0.01), whereas ABTS showed lower correlation coefficients with FRAP (r = 0.456, *p* < 0.05) and DPPH (r = 0.595, *p* < 0.01). TPC was significantly correlated with DPPH (r = 0.839, *p* < 0.01), ABTS (r = 0.734, *p* < 0.01), and FRAP (r = 0.683, *p* < 0.01), aligning with the study of Escuredo et al. [73].

These variations in correlation may be attributed to the differences in the underlying mechanisms of each assay. The DPPH assay, which measures the scavenging activity of antioxidants on a stable organic radical, is more applicable to hydrophobic systems, while the ABTS assay involves a radical cation (ABTS^+^) that is reactive in both hydrophilic and lipophilic environments [79]. In contrast, the FRAP assay does not involve free radicals but instead measures the reduction of ferric iron (Fe^3+^) to ferrous iron (Fe^2+^), primarily reflecting the reducing power of the sample rather than the radical scavenging capacity [80]. These methodological differences may explain the observation variations in correlation coefficients, as the antioxidant activity of SBH is influenced by its composition [81].

### 3.3. Antimicrobial Activity

#### 3.3.1. Agar Diffusion Assay

The agar diffusion assay results (Table 5) showed that all SBH samples exhibited inhibitory activity against *S. aureus*, whereas only six *T. binghami* honey samples demonstrated inhibition against *E. coli*. Among the three species, *T. binghami* honey exhibited the strongest inhibitory activity, with an average inhibition zone of 20.18 mm against *S. aureus*, significantly larger than that of *H. itama* honey (11.92 mm) and *L. canifrons* honey (11.59 mm). These results indicate that the antimicrobial properties of SBH vary by bee species and bacteria strain. Our findings are consistent with those of Cabezas-Mera et al. [12], who reported that the antimicrobial properties of SBH are influenced not only by stingless bee species and the bacteria stain but also by honey concentration, incubation time, and the bioactive compounds of honey. Notably, our results align with previous research showing that SBH exhibits stronger inhibitory activity against Gram-positive bacteria than Gram-negative bacteria [82]. Additionally, certain stingless bee honey samples have been found to have no inhibitory effects against *E. coli* [83]. Future studies should take into account additional factors such as honey concentration and incubation time to better elucidate the mechanisms underlying its antibacterial activity.

**Figure 1:**
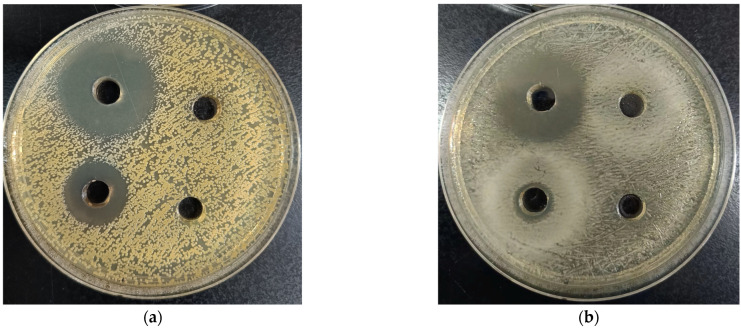
Antibacterial effects of different honeys on (**a**) *S. aureus* and (**b**) *E. coli*. Note: In the culture medium, top left: positive control, top right: catalase-treated honey solution, bottom left: 50% (*w*/*v*) honey solution and bottom right: negative control; (**a**) HI 4 SBH sample, (**b**) TB 6 SBH sample.

Hydrogen peroxide is known to be a key factor contributing to the antibacterial activity in honey [71]. To investigate the primary antibacterial component of SBH, catalase-treated honey solutions were subjected to the same assay. Partial results are presented in Figure 1. None of the experimental groups exhibited inhibition zones, suggesting that hydrogen peroxide is the primary antibacterial component in honey [84,85]. Interestingly, Jibril et al. [86] observed that certain SBH samples still produced inhibition zones against *S. aureus* even after being treated with catalase. This indicates the presence of non-peroxide antibacterial components in stingless bee honey, consistent with the findings of Saputra et al. [87] and Wu et al. [64], which showed that honey contains other antibacterial components such as organic acids, phenolic compounds, flavonoids, terpenoids, and alkaloids.

#### 3.3.2. Broth Microdilution Assay

The effectiveness of the agar diffusion assay depends on the diffusion capacity of honey, which may limit its ability to fully express antibacterial potential [88]. In contrast, the microdilution assay enables direct interaction between the antibacterial components and microorganisms, providing a more accurate evaluation of antibacterial activity [89]. Therefore, employing this method is essential for a comprehensive assessment of honey’s antibacterial properties.

The MBC values determined by the broth microdilution assay ranged from 3.125% to 20% (Table 6). In the *H. Itama* (HI) group, MBC values against *S. aureus* ranged from 5% to 12.5%, with HI 4 exhibiting the lowest MBC, demonstrating the strongest bactericidal activity within the group. Interestingly, the HI honey in this study demonstrated greater bactericidal effects compared to the findings of Tuksitha et al. [90]. For the *L. canifrons* (LC) group, the MBC values against *S. aureus* ranged from 6.25% to 20%, with LC 2 showing the strongest bactericidal activity. Among the three species, the *T. binghami* (TB) group demonstrated the most potent antibacterial effects, with the MBC values against *S. aureus* ranging from 3.125% to 6.25%.

Overall, TB 6 demonstrated the strongest antibacterial activity in the broth microdilution assay, consistent with the agar diffusion assay results. This may be attributed to its low pH value (2.83). All the samples exhibited stronger antibacterial activity in *S. aureus* than *E. coli*. This aligns with the research from Eloi de Sousa Guimarães et al. [91], who discovered that SBH exhibited better inhibitory and bactericidal effects against *S. aureus*, with higher moisture content enhancing antibacterial activity. Additionally, the presence of specific probiotics in SBH, which produce antibacterial compounds, likely contributes to its antibacterial properties [92].

#### 3.3.3. Correlation Coefficients Between Antimicrobial Activities and Antioxidant Activities

To investigate the relationship between antimicrobial and antioxidant activities, a correlation analysis was conducted (Table 7). A significant correlation was observed between MBC (*S. aureus*) and MBC (*E. coli*) (r = 0.790, *p* < 0.01). While TPC and FRAP did not show a significant correlation with antimicrobial activity, ABTS exhibited a significant correlation with both MBC (*S. aureus*) and MBC (*E. coli*). Although Tuksitha et al. [90] reported that the antibacterial activity of honey is affected by phenolic and flavonoid compounds, our results align with Bueno-Costa et al. [43], who found no correlation between TPC and antibacterial activity. These results suggest that certain antioxidant compounds may contribute to the antimicrobial properties.

#### 3.3.4. Correlation Between Bioactivities and Physicochemical Parameters

The correlation between bioactivities and physicochemical parameters was analyzed (Table 8). TPC showed a significant negative correlation with moisture content (r = −0.467) but exhibited a significant positive correlation with color (r = 0.716), trehalulose content (r = 0.498), and pH (r = 0.410). Consistent with these findings, studies by Yap et al. [93] and Wu et al. [64] reported that the TPC in stingless bee honey increased after dehydration, accompanied by a darker color. Additionally, all antioxidant-related parameters exhibited significant positive correlations with color, indicating that darker honey is associated with stronger antioxidant properties, in agreement with the findings of Pita-Calvo and Vázquez [48]. ABTS also displayed significant negative correlations with moisture content, EC, and acidity.

Furthermore, MBC for *S. aureus* and *E. coli* demonstrated significant positive correlations with trehalulose content and pH. Brown et al. [94] previously reported that low pH in SBH is generally associated with enhanced antibacterial activity. However, Zhang et al. [88] found an opposite trend in honeybee honey, where higher pH values correlated with stronger antimicrobial effects. This discrepancy suggests that the relationship between pH and antimicrobial activity may be species-dependent and influenced by the complex physicochemical composition of honey. Additionally, significant negative correlations were found between MBC (for both *S. aureus* and *E. coli*) and moisture content, EC, and acidity, indicating that higher values of these parameters correspond to lower MBC values, reflecting stronger antibacterial properties. However, our findings are not entirely consistent with those of Hossain et al. [89], who suggested that acidity, a low water content, and a high sugar content enhance the antimicrobial activity of honey. These results highlight the complexity of the antimicrobial mechanism in SBH, suggesting that its efficacy is influenced by multiple physicochemical factors rather than a single parameter.

## 4. Conclusions

This study investigated the physicochemical characteristics, antioxidant capacity, and antimicrobial activity of Malaysian stingless bee honey, highlighting the distinct properties among different species. The results demonstrate that *L. canifrons* honey exhibits the highest trehalulose content, while *H. itama* honey exhibits the strongest antioxidant capacity. Notably, *T. binghami* honey displays the most potent antimicrobial effects, which were associated with its highest moisture content, electricity conductivity, acidity, and lower pH. Furthermore, a strong correlation was observed between the honey’s ABTS antioxidant activity and its moisture content, suggesting that moisture-related factors may influence antioxidant potential. Among the physicochemical parameters, the strongest correlation was observed between EC and acidity. Additionally, the MBC values against *S. aureus* and *E. coli* showed the strongest correlation with the bioactivities of honey, emphasizing the potential of stingless bee honey as a natural antimicrobial agent. These findings underscore the unique properties of stingless bee honey across different species. Further research is warranted to better characterize these differences and their underlying mechanisms. Additionally, this study provides valuable data to support the development of international quality standards for stingless bee honey and offers insights into its potential health benefits.

## Figures and Tables

**Table 1:** Details of stingless bee honey samples.

Honey	Sample	Harvest Time	Location of Bee Farm	Botanical Origin
*H. itama*	HI 1-HI 3	2023.8	Labuan	Fruit trees, forest
	HI 4	2023.8	Labuan	*Acacia mangium*, star fruit, rambutan, wild vegetation
	HI 5	2023.8	Skudai, Johor	Fruit trees, forest
	HI 6	2023.8	Temiang, Negeri Sembilan	*A. mangium*, banana, wild vegetation
	HI 7	2022.12	Temiang, Negeri Sembilan	*A. mangium*, banana, wild vegetation
	HI 8-HI 9	2023.6	16 miles, Sarawak	*A. mangium*, wild vegetation
	HI 10	2022.11	16 miles, Sarawak	*A. mangium*, wild vegetation
	HI 11	2023.6	Panasu, Sarawak	fruit trees, forest
*L. canifrons*	LC 1-LC 3	2023.8	Temiang, Negeri Sembilan	*A. mangium*, banana, wild vegetation
	LC 4-LC 6	2023.7	Gambang, Pahang	*A. mangium*, fruit trees
	LC 7-LC 9	2023.8	Bangi, Selangor	Coconut trees, plants
*T. binghami*	TB 1-TB 3	2023.8	Ng. Dap, Sarawak	*A. mangium*, wild vegetation
	TB 4-TB 8	2023.7	Ng. Dap, Sarawak	*A. mangium*, wild vegetation
	TB 9	2022.8	Jelebu, Negeri Sembilan	fruit trees, forest
	TB 10	2022.12	Jelebu, Negeri Sembilan	fruit trees, forest

HI: *H. itama* honey; LC: *L. canifrons* honey; TB: *T. binghami* honey.

**Table 2:** Physicochemical characteristics of three Malaysian stingless bee honey.

Parameters	Stingless Bee Species		
	*H. itama* (*n* = 11)	*L. canifrons* (*n* = 9)	*T. binghami* (*n* = 10)
Moisture (%)	29.05 ± 1.02 a	27.61 ± 2.59 a	34.67 ± 2.29 b
	(27.12–30.48)	(25.44–33.45)	(32.78–40.36)
Color (mm)	105.09 ± 32.17 a	77.22 ± 36.07 ab	62.60 ± 38.85 b
	(64.00–150.00)	(31.00–140.00)	(31.00–150.00)
Fructose (g/100 g)	11.28 ± 3.59 a	7.60 ± 1.20 b	9.80 ± 3.93 ab
	(6.17–16.91)	(5.66–9.31)	(5.45–15.74)
Glucose (g/100 g)	11.50 ± 5.00 a	5.49 ± 1.40 b	17.09 ± 3.54 c
	(5.61–19.45)	(3.85–7.76)	(7.31–19.64)
Sucrose (g/100 g)	0.70 ± 0.72 a	0.73 ± 0.82 a	1.37 ± 0.36 a
	(ND-1.89)	(ND-2.47)	(0.68–1.90)
Trehalulose (g/100 g)	33.59 ± 7.12 a	38.13 ± 7.38 a	20.81 ± 3.52 b
	(22.71–41.77)	(22.64–43.75)	(15.42–24.75)
5-HMF (mg/kg)	1.33 ± 0.72 a	2.03 ± 1.80 a	1.69 ± 0.68 a
	(0.53–2.63)	(ND-5.40)	(0.53–2.44)
Diastase activity (DN)	ND	ND	ND
pH	3.17 ± 0.12 a	3.21 ± 0.16 a	2.83 ± 0.17 b
	(2.99–3.36)	(2.93–3.39)	(2.62–3.09)
Electricity Conductivity (μs/cm)	810.73 ± 134.33 a	608.17 ± 141.48 a	1005.28 ± 285.88 ab
	(578.25–1066)	(478.40–889.40)	(788.70–1573.00)
Acidity (meq/kg)	238.57 ± 47.61 a	305.69 ± 70.66 a	515.72 ± 146.48 b
	(140.99–312.78)	(240.85–446.17)	(395.90–787.89)

Results are expressed as mean ± standard deviation and (minimum–maximum). Mean values within a row that share the same letter are not significantly different for *p* < 0.05. “ND” means not detected.

**Table 3:** Correlation coefficients between physicochemical parameters.

	Moisture	Color	Trehalulose	pH	EC	Acidity	5-HMF
Moisture	1	−0.155	−0.668 **	−0.570 **	0.622 **	0.708 **	−0.141
Color	−0.119	1	0.158	0.132	0.467 **	0.117	−0.078
Trehalulose	−0.668 **	0.205	1	0.738 **	−0.569 **	−0.562 **	0.011
pH	−0.570 **	0.187	0.738 **	1	−0.501 **	−0.616 **	−0.177
EC	0.622 **	0.435 *	−0.569 **	−0.501 **	1	0.797 **	0.084
Acidity	0.708 **	0.096	−0.562 **	−0.616 **	0.797 **	1	0.232
5-HMF	−0.141	0.063	0.011	−0.177	0.084	0.232	1
Moisture	1	−0.155	−0.668 **	−0.570 **	0.622 **	0.708 **	−0.141

** Correlation is significant at *p* < 0.01; * correlation is significant at *p* < 0.05.

**Table 4:** Antioxidant activity of honey samples.

Stingless Bee Species	Total Phenolic Content (µg GAE/g)	DPPH (mg TE/100 g)	ABTS (mmol TE/kg)	FRAP (mmol FeSO_4_/kg)
*H. itama * (*n* = 11)	601.02 ± 143.43 a	21.23 ± 10.25 a	3.67 ± 0.46 a	2.33 ± 0.70 a
	(424.43–880.23)	(10.63–42.48)	(3.09–4.70)	(1.39–3.67)
*L. canifrons* (*n* = 9)	626.32 ± 70.91 a	17.84 ± 5.14 ab	3.53 ± 0.19 a	1.46 ± 0.64 b
	(531.89–730.97)	(7.93–23.26)	(3.16–3.74)	(0.42–2.31)
*T. binghami* (*n* = 10)	434.82 ± 102.00 b	10.80 ± 6.87 b	2.69 ± 0.27 b	1.44 ± 0.68 b
	(320.34–623.38)	(6.06–28.32)	(2.24–2.92)	(0.82–2.99)

Results are expressed as mean ± standard deviation and (minimum–maximum). Mean values within the three groups that share the same letter are not significantly different for *p* < 0.05.

**Table 5:** Inhibition zone diameters of honey samples against microorganisms using the agar diffusion method.

Samples	Inhibition Zones (mm)	
	*S. aureus*	*E. coli*
10% phenol solution	27.09 ± 0.99	23.81 ± 0.88
Sterile water	-	-
HI 1	9.31 ± 0.37	-
HI 2	8.73 ± 0.06	-
HI 3	8.26 ± 0.41	-
HI 4	18.62 ± 0.89	-
HI 5	8.29 ± 0.20	-
HI 6	10.12 ± 1.43	-
HI 7	8.05 ± 0.21	-
HI 8	17.34 ± 2.32	-
HI 9	18.58 ± 0.89	-
HI 10	14.94 ± 1.77	-
HI 11	8.97 ± 0.88	-
LC 1	13.82 ± 0.26	-
LC 2	18.56 ± 1.78	-
LC 3	8.92 ± 0.20	-
LC 4	9.31 ± 0.48	-
LC 5	9.44 ± 0.65	-
LC 6	19.59 ± 0.67	-
LC 7	8.14 ± 0.21	-
LC 8	8.32 ± 0.22	-
LC 9	8.24 ± 0.30	-
TB 1	22.58 ± 1.10	8.34 ± 0.31
TB 2	22.71 ± 1.52	8.75 ± 1.04
TB 3	23.72 ± 0.24	8.28 ± 0.48
TB 4	23.53 ± 0.37	9.28 ± 0.22
TB 5	22.64 ± 0.48	9.51 ± 0.51
TB 6	22.76 ± 0.21	10.09 ± 1.33
TB 7	17.28 ± 0.93	-
TB 8	18.14 ± 0.63	-
TB 9	14.06 ± 0.95	-
TB 10	14.39 ± 0.80	-
Mean for HI	11.92 ± 4.45 a	-
Mean for LC	11.59 ± 4.69 a	-
Mean for TB	20.18 ± 3.83 b	-

HI: *H. itama* honey; LC: *L. canifrons* honey; TB: *T. binghami* honey. Results are expressed as mean ± standard deviation. “-”: no zone of Inhibition. Mean values within the three groups that share the same letter are not significantly different for *p* < 0.05.

**Table 6:** The MBC values of honey samples against bacteria.

Honey	MBC/% (*w*/*v*)	
	*S. aureus*	*E. coli*
HI 1	10	20
HI 2	10	12.5
HI 3	12.5	20
HI 4	5	12.5
HI 5	12.5	20
HI 6	12.5	20
HI 7	12.5	25
HI 8	10	12.5
HI 9	10	20
HI 10	10	12.5
HI 11	10	12.5
LC 1	20	20
LC 2	6.25	12.5
LC 3	10	20
LC 4	10	20
LC 5	10	12.5
LC 6	10	12.5
LC 7	12.5	20
LC 8	12.5	20
LC 9	10	20
TB 1	5	10
TB 2	3.125	10
TB 3	5	10
TB 4	5	10
TB 5	3.125	10
TB 6	3.125	6.25
TB 7	6.25	10
TB 8	6.25	12.5
TB 9	5	10
TB 10	5	10

HI: *H. itama* honey; LC: *L. canifrons* honey; TB: *T. binghami* honey.

**Table 7:** Correlation between antimicrobial activities and antioxidant activities.

	MBC (*S. aureus*)	MBC (*E. coli*)
TPC	0.306	0.360
DPPH	0.332	0.445 *
ABTS	0.558 **	0.550 **
FRAP	0.137	0.255
MBC (S. aureus)	1	0.790 **
MBC (E. coli)	0.790 **	1

** Correlation is significant at *p* < 0.01; * correlation is significant at *p* < 0.05.

**Table 8:** Correlation between bioactivities and physicochemical parameters.

	Moisture	Color	Trehalulose	pH	EC	Acidity	5-HMF
TPC	−0.467 **	0.716 **	0.498 **	0.410 *	−0.057	−0.234	0.029
DPPH	−0.327	0.704 **	0.351	0.322	0.079	−0.188	−0.108
ABTS	−0.734 **	0.459 *	0.578 **	0.656 **	−0.430 *	−0.727 **	−0.101
FRAP	−0.142	0.731 **	0.069	0.059	0.382 *	−0.011	−0.087
MBC (*S. aureus*)	−0.707 **	0.134	0.663 **	0.671 **	−0.467 **	−0.601 **	−0.09
MBC (*E. coli*)	−0.619 **	0.052	0.607 **	0.641 **	−0.543 **	−0.635 **	0.029

** Correlation is significant at *p* < 0.01; * correlation is significant at *p* < 0.05.

## Data Availability

The original contributions presented in the study are included in the article, further inquiries can be directed to the corresponding author.
